# Premating isolation is determined by larval rearing substrates in cactophilic *Drosophila mojavensis*. X. Age-specific dynamics of adult epicuticular hydrocarbon expression in response to different host plants

**DOI:** 10.1002/ece3.1088

**Published:** 2014-04-23

**Authors:** William J Etges, Cassia C de Oliveira

**Affiliations:** Department of Biological Sciences, Program in Ecology and Evolutionary Biology1 University of Arkansas, Fayetteville, Arkansas, 72701

**Keywords:** Aging, cactus, cuticular hydrocarbons, desert, ethanol vapor, sexual isolation, sexual selection

## Abstract

Analysis of sexual selection and sexual isolation in *Drosophila mojavensis* and its relatives has revealed a pervasive role of rearing substrates on adult courtship behavior when flies were reared on fermenting cactus in preadult stages. Here, we assessed expression of contact pheromones comprised of epicuticular hydrocarbons (CHCs) from eclosion to 28 days of age in adults from two populations reared on fermenting tissues of two host cacti over the entire life cycle. Flies were never exposed to laboratory food and showed significant reductions in average CHC amounts consistent with CHCs of wild-caught flies. Overall, total hydrocarbon amounts increased from eclosion to 14–18 days, well past age at sexual maturity, and then declined in older flies. Most flies did not survive past 4 weeks. Baja California and mainland populations showed significantly different age-specific CHC profiles where Baja adults showed far less age-specific changes in CHC expression. Adults from populations reared on the host cactus typically used in nature expressed more CHCs than on the alternate host. MANCOVA with age as the covariate for the first six CHC principal components showed extensive differences in CHC composition due to age, population, cactus, sex, and age × population, age × sex, and age × cactus interactions. Thus, understanding variation in CHC composition as adult *D. mojavensis* age requires information about population and host plant differences, with potential influences on patterns of mate choice, sexual selection, and sexual isolation, and ultimately how these pheromones are expressed in natural populations. Studies of drosophilid aging in the wild are badly needed.

## Introduction

Studies of cuticular hydrocarbons (CHCs) serving as contact pheromones in insects have revealed a wealth of information concerning their biosynthesis (Schal et al. 1998; Howard and Blomquist 2005), regulation by a handful of genes (Dallerac et al. 2000; Gleason et al. 2005), diversity in closely related species (Page et al. 1997; Oliveira et al. 2011; Schwander et al. 2013), roles in waterproofing and transcuticular water loss (Gibbs and Pomonis 1995; Gibbs and Rajpurohit 2010), and sensitivity to biotic and abiotic factors. As pheromones, these molecules are involved in species and sex-specific recognition (Singer 1998) and mating status (Everaerts et al. 2010) during courtship and have been shown to mediate female preference by sexual selection (Chenoweth and Blows 2005; Havens and Etges 2013) and sexual isolation (Coyne and Charlesworth 1997; Etges and Ahrens 2001; Ishii et al. 2001; Dopman et al. 2004; Peterson et al. 2007) between populations and species. However, expression of CHCs has been shown to be plastic, modulated by temperature (Toolson 1982; Savarit and Ferveur 2002), humidity (Rouault et al. 2004), photoperiod (Krupp et al. 2008), rearing substrates (Etges 1992; Liang and Silverman 2000; Fedina et al. 2012; Kühbandner et al. 2012), and presence of other individuals or “social” effects (Kent et al. 2008; Krupp et al. 2008; Etges et al. 2009; Thomas and Simmons 2011), suggesting these chemical signals are highly context dependent and are another example of how flexible animal male–female signaling systems can be (Tinghitella et al. 2013). In most insects, CHCs are usually part of multimodal signaling systems where male–female physical contact precedes copulation. In many *Drosophila* species, CHC sensing occurs after male courtship movement, wing vibration, or song production, and involves male foreleg “rubbing” of the female's ventral abdomen and licking where males extend their proboscis in contact with a female's external genitalia prior to attempted copulation (Greenspan and Ferveur 2000).

In most cases, studies of *Drosophila* CHC expression have been performed under controlled laboratory conditions in order to minimize variation due to nutrition, sexual maturity, temperature, etc. Rearing substrates in particular are known to influence adult courtship behavior by influencing CHC composition (Etges and Ahrens 2001; Rundle et al. 2005; Etges et al. 2009). However, a full understanding of environmentally sensitive mating behaviors requires knowledge of conditions experienced by adults under natural conditions that contribute to differences in courtship success (see e.g. Grace et al. 2010). Here, we report the first attempt at characterizing CHC variation in adult *D. mojavensis* from eclosion to 28 days of age in flies exposed only to natural conditions, that is, fermenting cactus tissues like those used in nature.

### A brief description of the cactus-desert-*Drosophila* system

Throughout the deserts and arid lands of the southwestern USA and northwestern Mexico, *D. mojavensis* is one of four endemic *Drosophila* species that use the fermenting tissues of columnar cacti to carry out their life cycles (Heed 1982). Populations of *D. mojavensis* use agria cactus, *Stenocereus gummosus*, in Baja California, the islands in the Gulf or California, and in a small patch in coastal Sonora. Mainland populations in Sonora, Sinaloa, and Arizona use organ pipe cactus, *S. thurberi*, their main host with infrequent use of sina cactus, *S. alamosensis* (Ruiz and Heed 1988). In the Mojave Desert in Southern California, California barrel cactus, *Ferocactus cylindraceus*, is the principal host, and an insular population of *D. mojavensis* uses *Opuntia* spp. on Santa Catalina Island near Los Angeles, California. Mainland Mexico populations of *D. mojavensis* diverged from a Baja California ancestral population approximately 230–270 kya and then the Mojave population diverged from mainland populations approximately 117–135 kya with no recurrent gene flow (Smith et al. 2012). Thus, use of agria cactus in Baja California is considered to be ancestral, and invasion of the mainland and then Southern California was facilitated by switching to alternate cactus hosts.

In drosophilids, we are not aware of any laboratory study designed to assess CHC variation under simulated natural conditions throughout the life cycle to investigate CHC-mediated courtship behavior. As a first step, we designed a rearing experiment for *D. mojavensis* in which all individuals were reared under controlled conditions of fermenting cactus throughout their life cycle and assessed CHC variation in geographically isolated populations both exposed to the fermenting tissues of two major host cacti as adults aged. As diet and effects of age have been previously shown to influence sexual attractiveness (Fedina et al. 2012), we set out to characterize variation in CHCs in cactus-reared adults over their life span. Analyses of sexual selection and sexual isolation between mainland and Baja population reared in these conditions are underway.

## Methods and Materials

### Husbandry and origin of stocks

A total of 465 *D. mojavensis* adults were collected over banana baits in Punta Prieta, Baja California, in January 2008, and 1264 baited adults plus 9 adults that emerged from sina, *S. alamosensis*, rots were collected in a coastal cactus dry forest in Las Bocas, Sonora in March 2009. All flies were returned to the laboratory and cultured on banana food in 35-mL shell vials at room temperature (Brazner and Etges 1993) until the cactus culture experiments began in September 2009.

### Growth conditions: egg to adult

Thousands of vial-reared adults from each population were introduced into separate population cages (12,720 cm^3^) for 7–10 days and allowed to choose mates. Eggs collected from these cages were reared to eclosion on banana food at moderate larval densities in half-pint bottles in an incubator programmed at 27°C during the day and 17° at night on a 14:10 LD cycle in order to ameliorate vial-to-vial variation in culture conditions. Adults were then transferred to 35-mL shell vials in small same sex groups containing banana food until they were sexually mature (8–10 days). Approximately 200 females and 200 males from each population were introduced into separate oviposition chambers and allowed to mate and oviposit for 10 h each day. Eggs were collected from a 5.5-cm-diameter petri dish containing agar–cactus–yeast media attached to each oviposition chamber and washed in sterile deionized water, 70% ethanol, and again in deionized water. Eggs were counted into groups of 200, transferred to a 1-cm^2^ piece of sterilized filter paper, and placed in bottles containing fermenting cactus tissue. Cactus cultures, 15 for each of the four combinations of population and cactus, were started by autoclaving plugged half-pint bottles containing 75 g of aquarium gravel covered with a 5.5-cm-diameter piece of filter paper. Then, 60 g of either agria or organ pipe tissues were added and autoclaved again at low pressure for 10 min. Once at room temperature, each culture was inoculated with 1 ml aqueous solutions of a pectolytic bacterium, *Erwinia cacticida* (Alcorn et al. 1991) and of a mixture of seven cactophilic yeasts: *Dipodascus starmeri*, *Candida sonorensis*, *C. valida*, *Starmera amethionina*, *Pichia cactophila*, *P. mexicana*, and *Sporopachydermia cereana*. All unhatched eggs were counted to allow calculation of egg-to-adult viability, and all eclosed adults from each replicate culture were counted daily allowing determination of egg-to adult development time. Adults were separated by sex and immediately transferred in same sex groups of 30 flies to vials containing fermenting cactus. Thus, adults were never exposed to laboratory food. All cultures were maintained in the incubator described above.

### Growth conditions: adults

Cactus media for rearing adults was designed to be used as a feeding substrate and thus was prepared differently than that used for culturing larvae. This media was prepared by blending 953 g agria or organ pipe cactus tissue, 486 mL deionized water, and 5 g agar. This media was autoclaved for 15 min, cooled, inoculated with bacteria and yeasts (see above), fermented in a 37°C incubator for a week, and then placed into individual 2.2-cm-diameter plastic barrel plugs (Alliance Express, Little Rock, AR) fitted into one end of autoclaved 25 × 95 mm glass tubes. An additional inoculating loop of bacteria and the seven cactophilic yeasts was added to the fermenting cactus tissue in each food cap to supplement nutrition. The other end of each tube was fitted with an empty plug with a 1.75 cm hole sealed with fine mesh to allow air circulation after adding 30 adult females or males. All culture tubes were placed into sealed desiccators containing 1 L of 4% ethanol from 8:00 am to 6:00 pm in the incubator described above allowing the adults to feed on ethanol vapor, a significant energy source for adults that extends longevity and increases egg production (Etges 1989; Etges and Klassen 1989). For the remaining 14 h each day, all tubes were removed from the desiccators to minimize condensation and kept in the incubator. New plugs containing fermenting cactus were replaced every 4 days.

### Analysis of epicuticular hydrocarbon variation

Adults were sampled for CHC extraction on the day of eclosion, that is, day zero, and thereafter at 3, 6, 10, 14, 18, 24, and 28 days of age. Few adults survive past 4 weeks under these conditions (Etges and Heed 1992; Jaureguy and Etges 2007). Other flies raised in these experimental conditions were used to assess transcriptome variation across the life cycle. Total epicuticular hydrocarbons were extracted after lights on in the morning by immersing each adult in hexane for 20 min in a 300-*μ*L glass vial insert (Microliter Analytical Supplies, Suwanee, GA), evaporating off all hexane in a 40°C heating block, and freezing each sample at −20°C until analysis. Individual CHC extracts were redissolved in 5 *μ*L of heptane containing a known amount of docosane (C_22_) as an internal standard. Each sample was analyzed by capillary gas–liquid chromatography using an automated Shimadzu GC-17A (Shimadzu Scientific Instruments, Columbia, MD) fitted with a flame ion detector (FID) and a 15 m (ID = 0.22 mm) Rtx-5 fused-silica column (Restek Corporation, Bellefonte, PA). Injector and detector temperatures were set at 290°C and 345°C, respectively, with the injector port in split mode (3:1 ratio), and the column was heated from 200°C to 345°C at 15°C/min holding at 345°C for 4 min.

Amounts of 31 CHC components (Stennett and Etges 1997; Etges and Ahrens 2001; Etges and Jackson 2001) were quantified in all flies by analysis of peak integrations using Class VP 4.2 software provided by Shimadzu, quantified using amounts of C_22_ as an internal standard, and expressed as nanograms/fly. All CHC data were log_10_-transformed to improve normality. We sampled five adults for each combination of population, cactus, and sex, and MANCOVA was used to quantify significant sources of variation in SAS using the model:


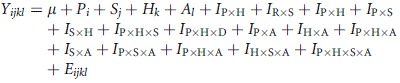


where *μ* is the grand mean, *P*_i_ is the effect of population (Baja California vs. the mainland), *S*_j_ is the effect sex, *H*_k_ is the effect of host cactus, *A*_l_ is the effect of age as a covariate, *I*_P×H_ is the interaction between population and cactus, *I*_P×S_ is the interaction between population and sex, *I*_S×H_ is the interaction between sex and cactus, *I*_P×H×S_ is the interaction between population, sex, and cactus, I_P×A_ is the interaction between population and age, I_H×A_ is the interaction between cactus and age, etc., and *E*_ijk_ is the error term. Principal components analysis (PCA) was used to identify different combinations of correlated CHC amounts and used in ANOVAs to assess overall sources of variation. All analyses were performed with SAS (SAS-Institute 2004). GC-MS identification of most of these CHCs was described in Toolson et al. (1990) and Etges and Jackson (2001).

## Results

We carefully monitored culture conditions to insure eclosed adult flies were of consistent size and fitness as in previous experiments. Estimates of egg-to-adult viability and development time (DEVT) of each population reared on either agria or organ pipe revealed greater viability (*F* = 10.26, *P* = 0.002, df = 1/56) of Punta Prieta, Baja California flies than Las Bocas, Sonora flies, 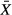 ± 1 SE, 78.8 ± 0.02 vs. 71.1 ± 0.02, respectively, and longer DEVT (*F* = 77.20, *P* < 0.0001, df = 1/112) of Las Bocas flies than Punta Prieta flies, 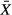 ± 1 SE, 16.45 ± 0.09 da vs. 15.38 ± 0.09 da, respectively. Cactus substrates had no effect on viability, but organ pipe caused longer DEVT (*F* = 148.65, *P* < 0.0001, df = 1/112) than agria cactus, 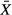 ± 1 SE, 16.65 ± 0.09 da vs. 15.19 ± 0.09 da, respectively, and there was a significant Population × Cactus interaction in DEVT (*F* = 4.85, *P* = 0.03, df = 1/112). Thus, our culture conditions produced adult flies similar to those in previous studies of cactus-reared Baja California and mainland populations (Etges 1990; Etges et al. 2010).

### Lifetime variation in CHCs

**Figure 1:**
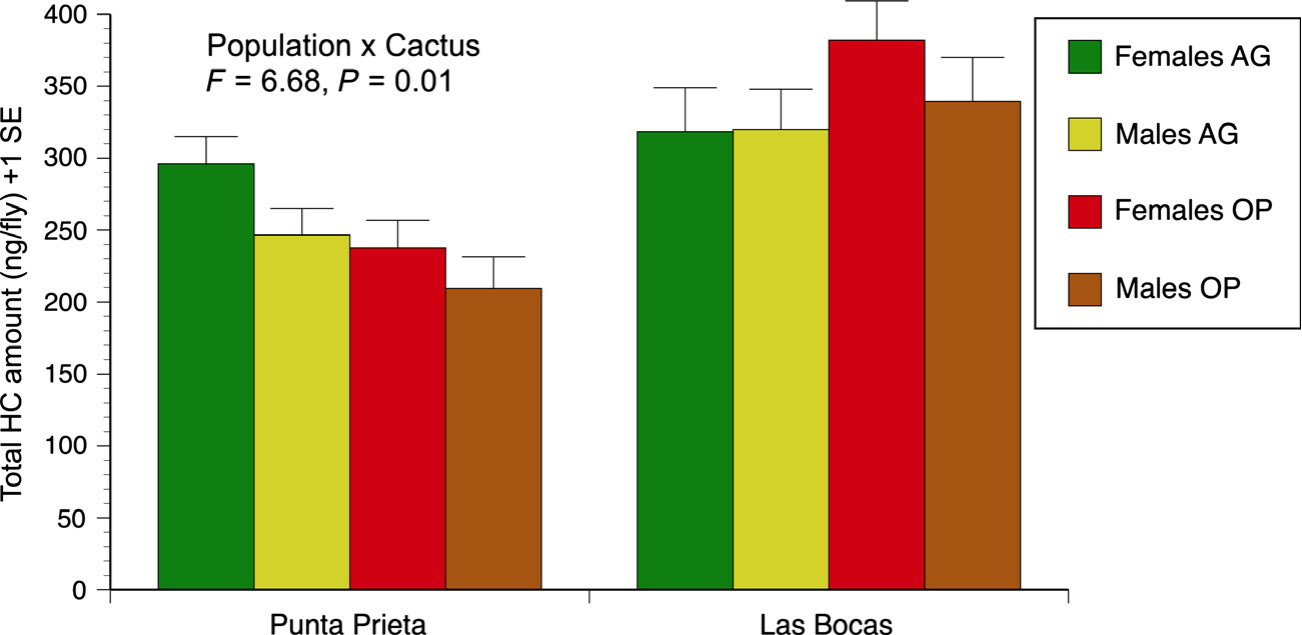
Total cuticular hydrocarbon amounts of male and female *Drosophila mojavensis* reared on agria and organ pipe cactus as adults revealing a population × cactus interaction from ANOVA.

In most cases, 3–5 adults from each treatment combination were included for analysis. Two groups, 14-day-old Las Bocas females reared on agria and 28-day-old Punta Prieta males reared on organ pipe, were not included due to insufficient sample sizes. Almost all model effects in the MANCOVA were statistically significant with population and age showing the largest F values (Table 1). CHC differences due to adult age also showed significant interactions with all of the other model terms emphasizing the sensitivity of lifetime CHC expression from eclosion onwards to differences in sex, population, and host plants. Punta Prieta adults produced more total CHCs when reared on their host plant, agria cactus, while Las Bocas adults expressed more CHCs when reared on their native host, organ pipe cactus (Fig. 1).

**Table 1:** MANCOVA results for cuticular hydrocarbon variation for adult male and female *Drosophila mojavensis* from 0 to 28 da of age reared on fermenting agria and organ pipe cactus

Source of variation	Wilk's *λ*	*F*^1^	*P*
Population	0.2664	17.41	<0.0001
Cactus	0.5793	4.59	<0.0001
Population × Cactus	0.7267	2.38	0.0002
Sex	0.7243	2.41	0.0002
Population × Sex	0.8480	1.13	0.298
Cactus × Sex	0.7857	1.72	0.014
Population × Cactus × Sex	0.8185	1.40	0.089
Age	0.2346	20.63	<0.0001
Age × Population	0.5497	5.18	<0.0001
Age × Cactus	0.6350	3.63	<0.0001
Age × Population × Cactus	0.6759	3.03	<0.0001
Age × Sex	0.6217	3.85	<0.0001
Age × Population × Sex	0.8053	1.53	0.045
Age × Cactus × Sex	0.8020	1.56	0.038
Age × Population × Cactus × Sex	0.7995	1.59	0.033

1 All df = 31,196.

### Major axes of CHC variation

**Figure 2:**
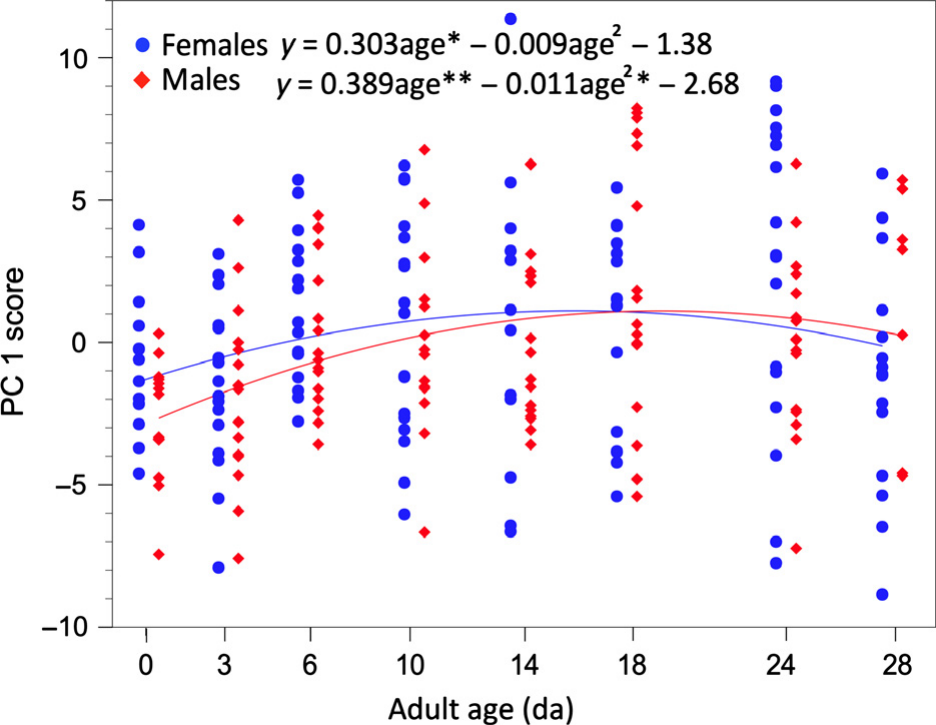
Age-specific shifts in adult cuticular hydrocarbons represented by variation principal component 1 from 0 to 28 days of age. Second-order regression equations are shown for each sex with populations and cactus pooled. *P < 0.05; **P < 0.01.

To untangle causal factors responsible for changes in CHCs with age, we used PCA to identify major components of variation in our data (cf. Etges and Ahrens 2001; Etges et al. 2009; Rajpurohit et al. 2013). The first six principal components accounted for 75.8% of the variation in the data, with PC1 accounting for 46.2% of the variance and PC6 only 3.2% (Table 2). Loadings on PC1 were uniformly positive suggesting that approximately half the variation in these data was due to overall up/down shifts in all CHC amounts due to biological causes and experimental error. Age trajectories in total CHCs amounts represented by PC1 were similar in both sexes and best approximated by curvilinear regressions (Fig. 2); higher-order terms were not significant. CHC amounts increased from eclosion to approximately 14–18 days of age and then decreased until 28 day similar to the profiles reported by Toolson et al. (1990), although they reported increases from eclosion to 10 days in a mainland population of laboratory food-reared flies maintained at slightly higher temperatures.

**Table 2:** Loadings of each hydrocarbon component on each of the six Principal Components in this study

Hydrocarbon^1^	ECL^2^	PC1	PC2	PC3	PC4	PC5	PC6
2-methyloctacosane	C_28.65_	0.225	0.019	−0.096	−0.175	0.109	−0.061
2-methyltricontane	C_30.65_	0.209	0.006	0.170	−0.064	0.125	−0.067
7- and 9-hentricontene	C_30.78_	0.155	0.121	0.304	−0.033	0.086	−0.014
Unknown	C_32_	0.111	0.057	−0.273	0.519	−0.013	−0.101
Unknown alkene	C_33br1_	0.178	−0.154	−0.065	0.044	0.147	−0.109
11- and 13-methyldotricontane	C_33br2_	0.220	−0.039	−0.075	−0.038	0.175	−0.241
Unknown alkene	C_33br3_	0.202	−0.155	−0.196	−0.050	0.315	−0.008
31-methyldotricont-8-ene	C_32.47_	0.182	−0.279	−0.032	−0.017	−0.352	−0.184
31-methyldotricont-6-ene	C_32.56_	0.121	0.107	0.057	0.056	0.675	0.123
8,24-tritricontadiene	C_32.63_	0.212	−0.016	0.020	−0.152	−0.259	−0.258
7,25-tritricontadiene	C_32.70_	0.161	0.045	0.189	−0.008	0.040	−0.331
10-, 12-, and 14-tritricontene	C_32.79_	0.192	−0.192	−0.134	−0.131	−0.026	0.247
Unknown	C_32.86_	0.145	−0.273	0.041	−0.019	−0.117	0.269
8,26-tetratricontadiene	C_34diene1_	0.204	0.164	−0.260	−0.095	−0.048	−0.042
6,24- and 6,26-tetracontadiene	C_34diene2_	0.191	0.197	−0.096	0.365	−0.095	−0.073
10-, 12-, and 14 tetretricontene	C_34ene_	0.167	−0.018	−0.133	0.441	0.063	0.098
33-methlytetratricont-10-ene	C_35alk1_	0.230	−0.129	−0.154	−0.079	0.124	−0.105
33-methlytetratricont-8-ene	C_35alk2_	0.221	−0.210	−0.106	−0.061	−0.083	0.061
Unknown alkene	C_35alk3_	0.235	−0.092	0.127	0.110	−0.031	−0.001
9,25-pentatricontadiene	C_34.59_	0.198	0.195	−0.263	−0.184	−0.093	−0.041
8,26-pentatricontadiene	C_34.66_	0.215	0.000	0.309	0.045	−0.031	0.035
7,27-pentatricontadiene	C_34.73_	0.120	0.116	0.240	0.148	−0.146	−0.123
Unknown diene	C_36a_	0.202	0.209	0.092	−0.006	−0.086	0.195
Unknown alkene	C_36b_	0.150	−0.040	0.142	0.318	−0.061	0.226
35-methylhexatricont-10-ene	C_37br_	0.077	0.049	0.304	−0.120	0.122	−0.400
9,27-heptatricontadiene	C_36.5_	0.196	0.070	−0.235	−0.237	−0.111	0.121
8,28-heptatricontadiene	C_36.6_	0.207	−0.121	0.121	−0.075	−0.020	0.215
14-, 16-, and 12-hexatricontene	C_36.7_	0.159	−0.155	0.344	0.066	−0.110	0.193
Unknown	C_38_	0.099	0.474	−0.014	0.005	−0.150	−0.033
Unknown	C_39_	0.129	0.413	−0.023	−0.065	−0.078	0.137
Unknown	C_40_	0.087	0.237	0.096	−0.204	0.050	0.369
Eigenvalue		14.333	2.792	2.374	1.666	1.316	0.984
Percentage of total variance		0.462	0.090	0.077	0.054	0.043	0.032

1 The 31 epicuticular hydrocarbon components in *D. mojavensis* included – most identified by GCMS; Etges and Jackson (2001), based on all adults in this study reared on both host cacti (*n* = 242).

2 Equivalent chain length based on relative retention times with known standards.

**Figure 3:**
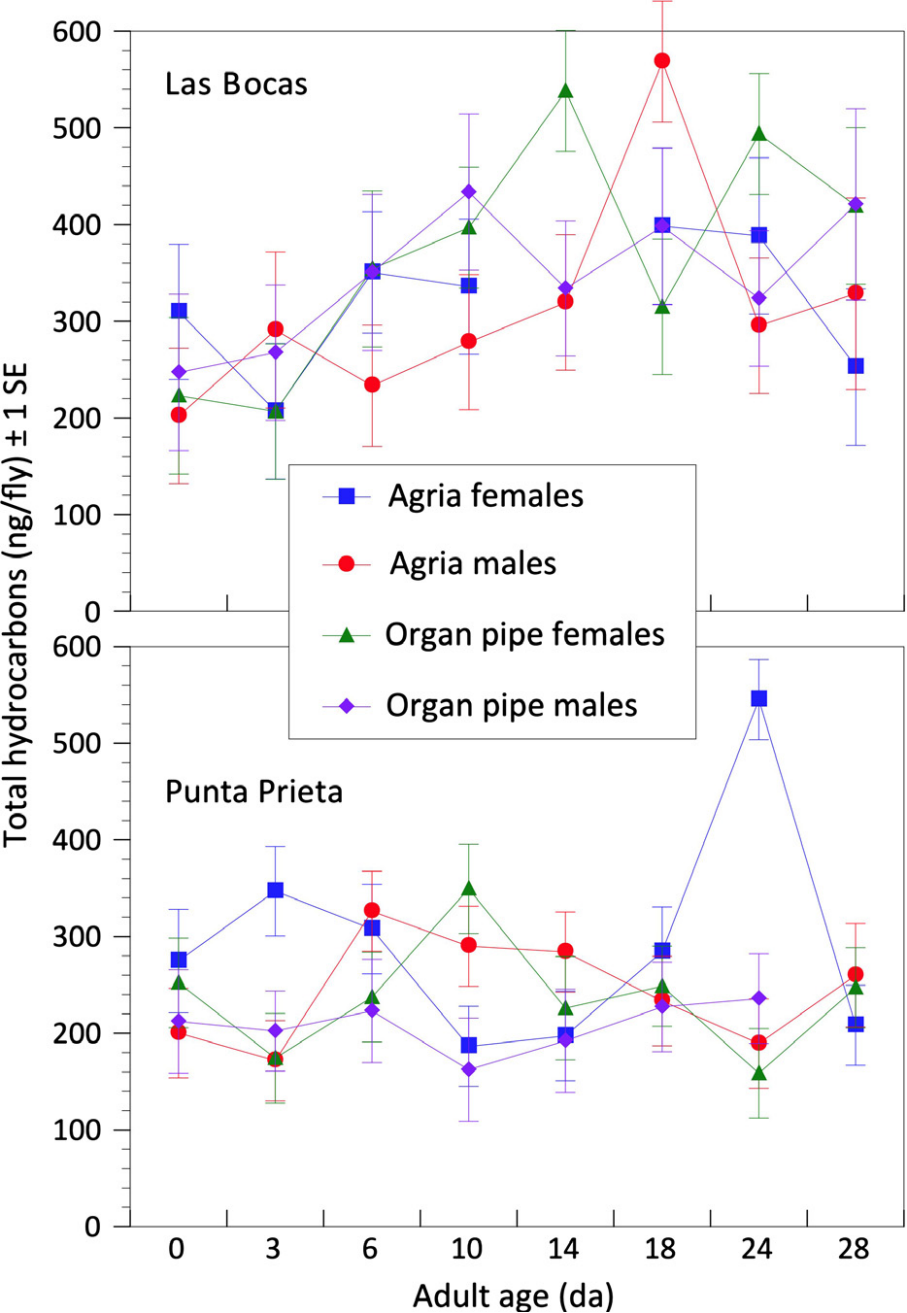
Plots of population, sex, and cactus specific changes in cuticular hydrocarbons from 0 to 28 days of age emphasizing population-specific differences in age-related shifts between these mainland and Baja California populations. See text for details.

**Figure 4:**
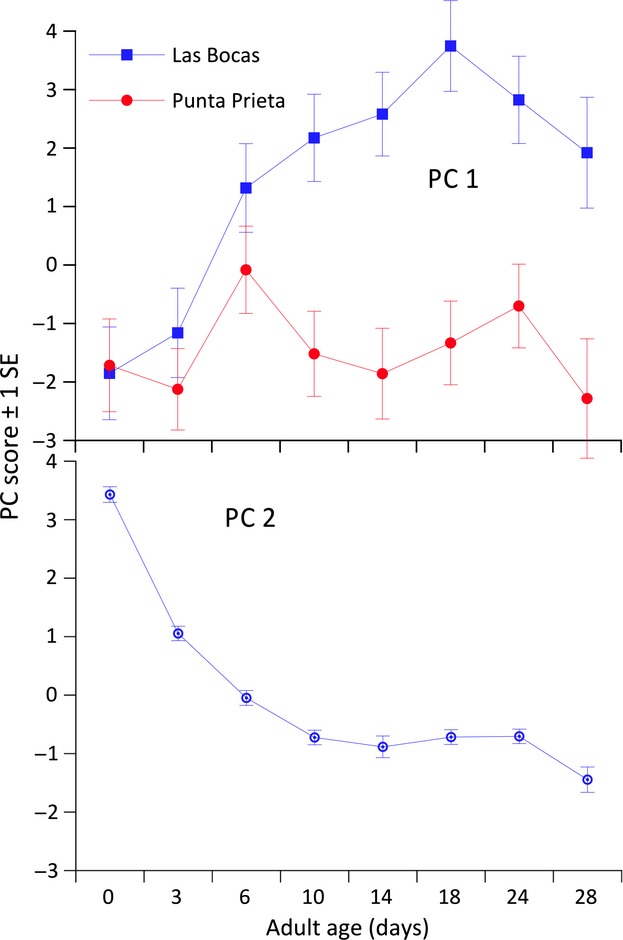
Cuticular hydrocarbon PC 1 scores with age contrasting population differences, and PC 2 plotted by age showing age-related increases in CHC abundance. See text for details.

In order to identify experimental sources of variation in CHCs, we used ANCOVA for each of the first six principal components to reveal variation not readily apparent from the PC loadings (Table 2). Only age and an age × population interaction were significant model effects for PC1 (Table 3), but plots of total CHCs per fly revealed significant differences among populations (Fig. 3) that were significant only for PC3, PC4, and PC6. Las Bocas adult total CHC amounts increased from eclosion to 18 days and then decreased until 28 days, but Punta Prieta CHCs increased from eclosion to 6 days and then decreased again (Figs. 3, 4). Thus, mainland and Baja California populations of *D. mojavensis* reared as adults on fermenting cactus and ethanol vapor exhibited significantly different (Table 1) lifetime profiles of CHC expression.

**Table 3:** ANCOVA results for the first 6 CHC Principal components for *Drosophila mojavensis* of increasing ages from two populations reared on agria and organ pipe cactus

	PC 1		PC 2		PC 3		PC 4		PC 5		PC 6	
						
Effect	F	Pr	F	Pr	F	Pr	F	Pr	F	Pr	F	Pr
Model	**5.20**^1^	**<0.0001**	**14.12**	**<0.0001**	**30.49**	**<0.0001**	**18.63**	**<0.0001**	**1.91**	**0.023**	**3.60**	**<0.0001**
Population	1.45	0.230	0.5	0.480	**89.66**	**<0.0001**	**19.85**	**<0.0001**	0.07	0.795	**15.78**	**<0.0001**
Cactus	0.24	0.627	0	0.998	**17.25**	**<0.0001**	0.08	0.781	0.06	0.808	0.47	0.494
Population × Cactus	0.31	0.579	0.01	0.933	3.3	0.071	0.14	0.705	2.59	0.109	0.31	0.576
Sex	2.12	0.147	1.56	0.213	**6.71**	**0.010**	0.2	0.655	2.40	0.123	1.25	0.265
Population × Sex	0.02	0.887	0.32	0.575	**4.05**	**0.045**	0.11	0.740	1.79	0.182	0.02	0.891
Cactus × Sex	0.23	0.630	0.75	0.388	0.23	0.635	2.63	0.106	3.56	0.061	3.27	0.072
Population × Cactus × Sex	0.7	0.405	0.01	0.920	0.55	0.460	0.62	0.432	2.19	0.141	0.55	0.459
Age	**11.85**	**0.001**	**168.5**	**<0.0001**	**41.04**	**<0.0001**	**32.72**	**<0.0001**	3.00	0.085	0.92	0.337
Age × Population	**9.61**	**0.002**	0.24	0.624	0.24	0.627	1.17	0.280	0.10	0.756	**29.38**	**<0.0001**
Age × Cactus	0.17	0.683	0.02	0.901	**22.95**	**<0.0001**	**9.61**	**0.002**	0.02	0.888	1.19	0.277
Age × Population × Cactus	1.27	0.262	0.94	0.333	2.25	0.135	1.65	0.200	3.17	0.076	0.5	0.478
Age × Sex	1.27	0.260	1.38	0.242	**12.56**	**0.001**	**36.00**	**<0.0001**	**7.64**	**0.006**	**5.47**	**0.020**
Age × Population × Sex	0.05	0.831	0.05	0.824	1.63	0.204	3.66	0.057	3.54	0.061	0.53	0.468
Age × Cactus × Sex	0.45	0.501	0.07	0.785	1.00	0.318	0.21	0.647	**5.70**	**0.018**	0.54	0.464
Age × Pop × Cactus × Sex	2.09	0.149	0.06	0.814	0.65	0.420	0.02	0.893	2.79	0.096	0.55	0.461

1 Significant effects are indicated in bold. Total *n* = 242, all df = 1,226.

Age differences accounted for variation in PC2 (*F* = 168.5, *P* < 0.0001; Table 3), so the structure of PC2 was of special interest in identifying individual CHCs most responsible for lifetime CHC shifts with age shared among populations. PC2 scores decreased in a curvilinear fashion from eclosion to a plateau extending from 10–24 days, and then decreased again at 28 days (Fig. 4), so negative loadings on PC2 (Table 2) indicated increases in CHC amounts and vice versa. Plots of the 16 most abundant CHCs with age in each population verified this (Figs. S1 and S2), but PC2+/− correlations with individual CHCs were not particularly large presumably due to the nonlinear shifts in CHC amounts with age and the differences between populations (Fig. 4). CHCs that increased in amounts with age, that is, those with large negative correlations with PC2, were C_33br1_, C_33br3_, 31-methyldotricont-8-ene, 10-, 12-, and 14-tritricontene, C_32.86_, 33-methlytetratricont-8-ene, 8,28-heptatricontadiene, and 14-, 16-, and 12-hexatricontene (Table 2). Those that decreased in abundance, that is, those with large positive correlations with PC2, were 7- and 9-hentricontene, 8,26-tetratricontadiene, 6,24- and 6,26-tetracontadiene, 9,25-pentatricontadiene, C_36a_, C_38_, C_39_, and C_40_. For both PC1 and PC2, the majority of CHC differences occurred between eclosion and day 10 consistent with Toolson et al. (1990).

**Figure 5:**
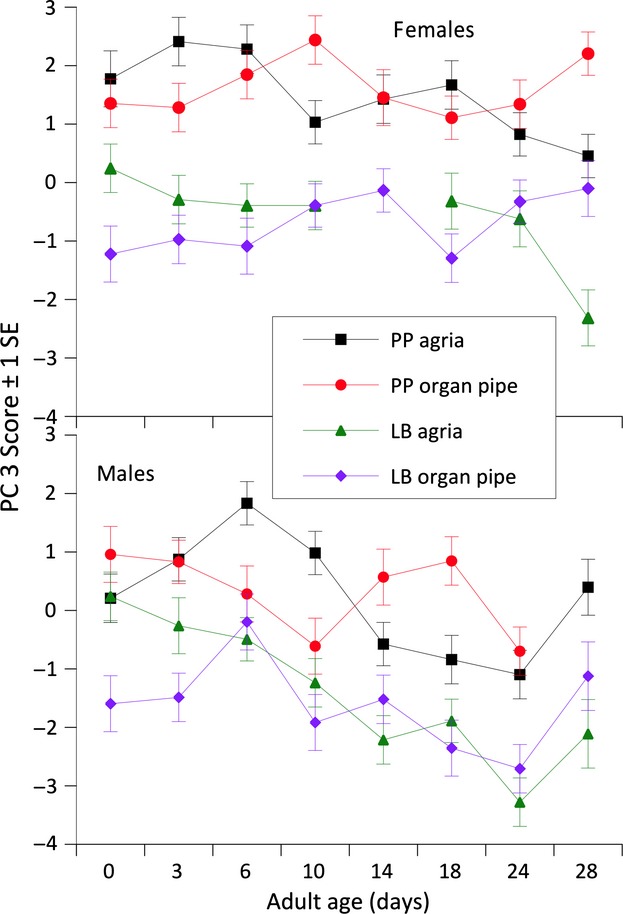
Age-specific variation in PC 3 scores showing differences due to sex, population, cactus, age × cactus, and age × sex interactions. See text for details.

CHC differences due to sex, population, and cactus influenced variation in PC3. These factors have long been known to influence regional variation in CHCs between Baja California and mainland populations of *D. mojavensis* (Etges and Ahrens 2001), but PC3 was also influenced by age, age × cactus, and age × sex interactions (Table 3). Thus, age-specific variation in CHC expression differed among sexes and shifted at different ages due to the type of fermenting cactus adults were exposed to (Fig. 5). Females showed less lifetime variation in PC3 scores than males, with the latter exhibiting decreasing PC3 scores particularly from 6 to 24 days. There was no significant interaction between population and cactus influencing PC3 or any higher-order interaction with age (Table 3), suggesting the effects of age on CHC expression were mainly dependent on the main effects of sex and host cactus. Groups of CHCs that were most highly correlated with PCs were those that differ geographically between Baja and mainland populations including both C_35_ alkadienes, 9,25-pentatricontadiene and 8,26-pentatricontadiene, as well as the C_37_ group, that is, 35-methylhexatricont-10-ene, 9,27-heptatricontadiene, and 8,28-heptatricontadiene. CHCs with higher positive loadings on PC3 tended to show significantly greater amounts in organ pipe cactus-reared flies and negative loading in agria-reared flies (Table 3), that is, 7- and 9-hentricontene, C_32_, C_32.86_, and 7,27-pentatricontadiene (all LSMEANS significantly different, *P* < 0.05, results not shown).

**Figure 6:**
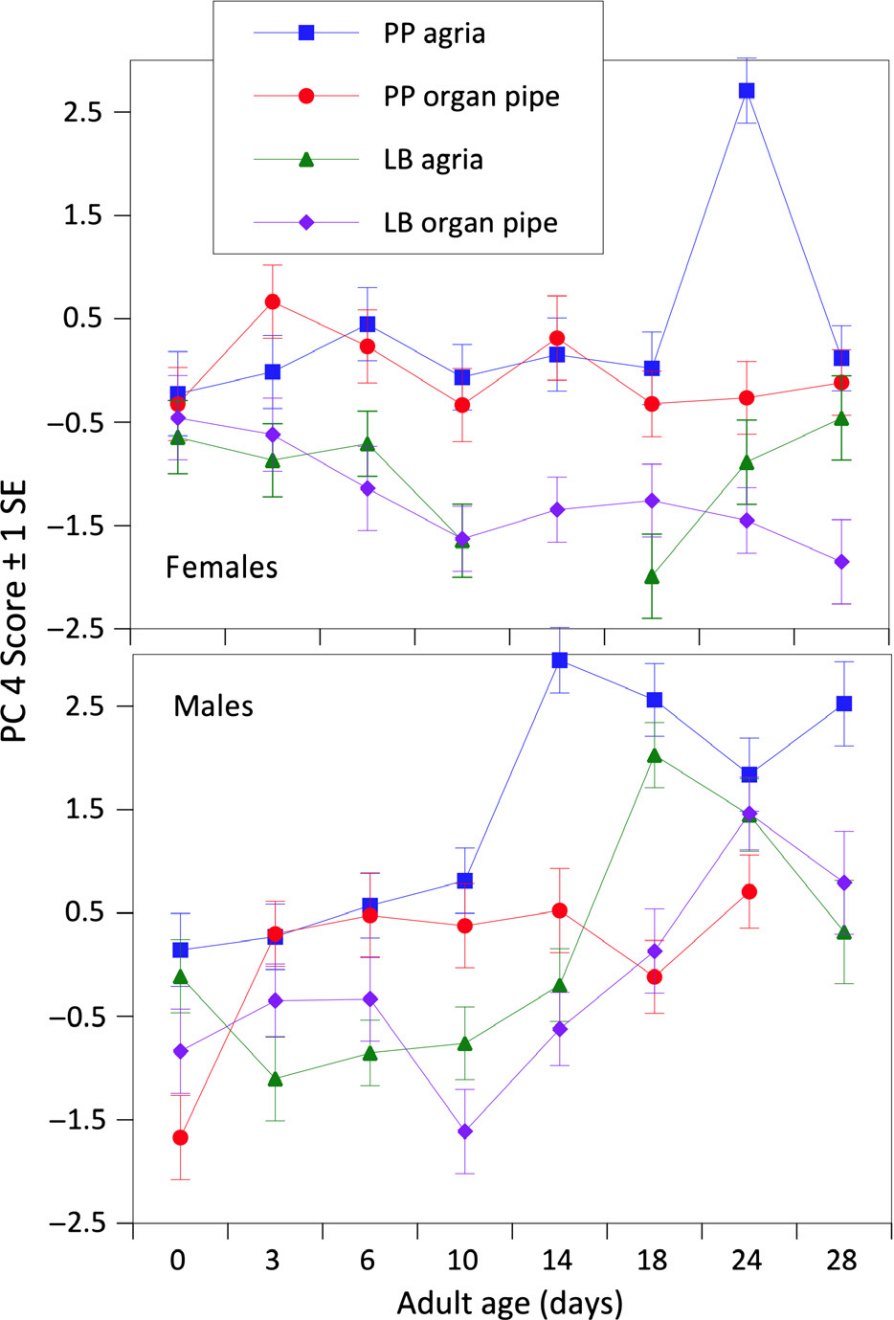
Male and female specific variation in PC 4 scores emphasizing an age × sex interaction in the changes in CHC expression with adult age.

Variation in PC4, accounting for 5.4% of the variation in the data, was influenced by most of the same model effects as PC3 indicating another independent (orthogonal) set of covarying CHCs was also influenced by population, age, age × sex, and age × cactus interactions, but not the main effect of sex (Table 3; Fig. 6). The age × sex interaction was caused by the clear decreases or unchanging PC4 scores with age in females and significant increases in PC4 scores in males (Fig. S3). Three CHCs were strongly positively correlated with PC4, that is, C_32_, 6,24- and 6,26-tetracontadiene, and 10-, 12-, and 14-tetretricontene, and each has been implicated with differences in mating success in both studies of sexual isolation (Etges and Tripodi 2008) and sexual selection (Havens and Etges 2013). In the latter study, mated males tended to have significantly less of these CHCs than unsuccessful males, suggesting these CHCs may be mating deterrents. These three CHCs increased in amounts with age, particularly at 18 days in Las Bocas males and 14- to 24-day-old Punta Prieta adults reared on agria (Figs. 1, 2).

Variation in PC5 and PC6 was influenced by fewer model effects, but these were mostly the same as the other PCs. Age × sex and age × cactus × sex interactions influenced PC5 that were most correlated with amounts of C_33br3_, 31-methyldotricont-8-ene, 31-methyldotricont-6-ene, 8,24-tritricontadiene, all C_33_ alkenes, and an alkadienes (Table 2). PC6 was influenced by population differences and population and sex interactions with age (Fig. S3), but there were few strong correlations with individual CHC components (Table 3). Overall, the changes in CHCs with age were dependent on sex, population, and host plants.

## Discussion

Identifying the causes of CHC expression in arthropods like *Drosophila* species has contributed to an understanding of chemical communication before and during courtship, including information exchange allowing identification of species, sex, and mating status (Howard and Blomquist 2005), as well as how efficiently adults control transcuticular water loss (Toolson 1978; Gibbs et al. 1998; Gibbs and Rajpurohit 2010). We found extensive variation in the expression of these molecules in *D. mojavensis* throughout adult life, documenting both increases and decreases in different combinations of CHCs from eclosion to age at first reproduction (AAFR) and then to age at death that was significantly influenced by population and host plant differences. Although total CHC amounts increased from eclosion onwards (Fig. 1), there was only weak correspondence between CHC amounts and AAFR in both populations (Fig. 3). Females attain sexual maturity before males, approximately 2–4 days for females and 5–10 days for males reared on laboratory food at 24–25°C (Markow 1982; Pitnick et al. 1995), but AAFR measured in adult females exposed to mature males, fermenting cactus, and ethanol vapor at 25°C was 5.2 and 5.3 da in mainland flies reared on agria and organ pipe cactus, respectively, and 7.3 and 5.8 da in Baja flies reared on agria and organ pipe cactus, respectively, with a population × cactus interaction (Etges and Klassen 1989). AAFR for males under natural conditions has yet to be assessed. Thus, it seems that CHCs convey information to the opposite sex concerning sex, host plant, and population differences that change with age, but not sexual maturity.

Significant differences in CHC composition were expected due to Baja versus mainland population and cactus effects (Etges and Ahrens 2001; Yew et al. 2011), but this is the first study to document variation in CHC profiles as adults aged under natural conditions and not exposed to laboratory media. Previous study of “ontogenetic variation” in adult *D. mojavensis* CHCs from a single mainland population reared on cornmeal–molasses laboratory media documented posteclosion increases in CHC abundance from 0- to 1-h-, 4-h-, 8-h-, 12-h-, 24-h-, 2-day-, 3-day-, 5-day-, 7-day-, 8-day-, and 21-day-old adults, with females showing larger lifetime increases than males, and peaks in total CHCs at approximately 8 da (Toolson et al. 1990). This trend was similar to overall shifts in total CHCs per fly for the mainland Las Bocas population and observed PC3 shifts (Fig. 5) suggesting that mainland populations may share this aging phenotype. Trends for male–female differences in individual alkanes, alkenes, and alkadienes in Toolson et al. (1990) were qualitatively similar to the lifetime shifts in the present study (Fig. S1), but direct comparisons were difficult due to the differences in sampling intervals.

### Aging, CHC variation, and behavior

Effects of aging on CHC profiles are of direct importance to understanding how male–female mating preferences change during adulthood, particularly given the roles of CHCs in sexual selection and sexual isolation between populations (Etges and Tripodi 2008; Etges et al. 2009; Havens et al. 2011; Havens and Etges 2013). CHC profiles continued to change with age after AAFR (Fig. 3), so causes other than signaling sexual maturity may be responsible for their age-dependent shifts in the context of sex, population, and host plant effects. As CHCs also mediate inhibition of water loss due to desiccation and temperature (Gibbs et al. 2003a,b; Rajpurohit et al. 2013), perhaps these age-specific profiles of CHC expression are the results of adaptation to desert conditions. If they are laboratory artifacts of housing flies in small, enclosed spaces in an incubator (see Toolson and Kuper-Simbron 1989), it will be necessary to survey age-specific CHC data from wild flies of known ages for comparison. Also, the present study was limited to hexane-soluble CHCs, so there may be other CHCs and triacylglycerides (Yew et al. 2011) expressed in an age-specific fashion that mediate male–female courtship interactions that were not included here.

While age-specific studies of CHC-related attractiveness in *D. mojavensis* have yet to be carried out, studies in other species have shown shifts in adult CHCs with age, for example, *D. virilis* (Jackson and Bartelt 1986) and shifts in attractiveness. Female *D. pseudoobscura* preferred mating with older males (Avent et al. 2008) and reduced sexual attractiveness in both sexes in very old (approximately 50 da) *D. melanogaster* was associated with increases in longer chain CHC amounts (Kuo et al. 2012b). The latter case involved increased expression of insulin pathway genes and possible involvement of the modulation of the target of rapamycin (TOR) pathway in older flies (Kuo et al. 2012a). Nutrient caused shifts in CHCs with age due to altered sugar and yeast amounts in laboratory food caused conflicting shifts in female attractiveness, suggesting laboratory environments may cause unpredictable changes in the signal properties of *D. melanogaster* CHCs (Fedina et al. 2012). Thus, changes in CHC profiles with age can influence mating preferences over the life span, at least in these laboratory studies.

### Laboratory versus nature: aging in the wild

An obvious question is: How long do *Drosophila* live in nature? Few data exist for wild adults in natural conditions, but laboratory studies of cactus-reared flies suggest > 90% of adults die by 30 days (Ganter et al. 1989; Etges and Heed 1992; Jaureguy and Etges 2007). Natural populations of *Opuntia*-breeding *D. mercatorum* and *D. hydei* exhibited daily survival rates of 0.81–0.97, but there was considerable year-to-year variation in mortality in Hawaiian populations (Johnston and Templeton 1982). By counting age layers on apodemes, internal muscle attachments (Johnston and Ellison 1982), adult age was determined for flies aged 0–16 days, but this technique is not reliable for flies older than this. Few individuals were captured that were 16 + days old suggesting most adults did not survive past approximately 2 weeks of age. Robson et al. (2006) compared median survivorship of laboratory *D. serrata* with predicted mean age of field-caught flies using eye pigment concentrations that changed with age. Laboratory-reared flies lived to an average of 30–40 days, while wild-caught flies had predicted ages of 6 days, with a range of 2–50 days. For a number of domesticated drosophilid species, short-term daily survival rates in natural populations were quite low, 0.45–0.85, corresponding to a mean adult life expectancy of 2.8 days leading the authors to conclude “This picture of survival is in stark contrast to most laboratory environments” (Rosewell and Shorrocks 1987). Thus, studies of aging in experimental populations of *Drosophila* under laboratory conditions bear little resemblance to patterns of adult survivorship in the wild.

### Laboratory vs. nature: CHCs in the wild

All attempts at comparing CHC variation across studies are compromised by differences in rearing conditions, particularly laboratory food vs. cactus (Brazner 1983; Stennett and Etges 1997). This is illustrated by perhaps the most surprising result from the present study: In most of our previous studies involving cactus-reared flies, adults reared on cactus through eclosion and then reared to maturity on laboratory food had 2–3 times as much CHCs, approximately 1000–1500 ng/fly (Etges and Ahrens 2001; Etges and Jackson 2001) as flies reared on fermenting cactus throughout the entire life cycle (Figs. 3, S1 and S2). In most of these previous studies, adults were aged to approximately 10–12 da to insure sexual maturity for mate preference tests. For comparison, total CHCs per fly for 14-day-old Las Bocas adults were 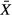 ± 1 SE; females, 538.2 ± 138.3 ng/fly, males, 326.7 ± 59.3 ng/fly; and for Punta Prieta adults, females, 209.5 ± 29.6 ng/fly, males, 249.6 ± 26.1 ng/fly (Fig. 3). Thus, laboratory food provides nutrients used to make much more CHCs from eclosion onward by adult *D. mojavensis* than fermenting cactus tissues and ethanol vapor even though flies were reared on fermenting cactus in preadult stages.

Further, our observations of CHC amounts in cactus + ethanol vapor-reared adults were consistent with the two known studies of CHCs from wild-caught *D. mojavensis*; (1) mainland adults from San Carlos, Sonora aspirated directly from organ pipe rots had much lower amounts of CHCs, 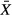 ± 1 SE; females, 418.0 ± 23.7 ng/fly, and males, 440.0 ± 23.0 ng/fly than laboratory food-reared flies (Toolson et al. 1990), and (2) wild-caught adults from three Baja and three mainland populations returned to the laboratory and exposed to laboratory food had less than half of total CHCs per fly than laboratory cactus-reared flies exposed to laboratory food as adults. Adults that eclosed from agria or organ pipe rots and reared to maturity on laboratory food had approximately 72% as much CHCs as cactus-reared flies exposed to laboratory food as adults (Etges 2002). Thus, as astutely suggested by Toolson et al. (1990), decreased amounts of CHCs in wild flies “suggests that compounds in *Stenocereus* tissue can directly affect synthesis and deposition of epicuticular hydrocarbons.” Certainly, laboratory food-reared adults have significantly more CHCs throughout life than flies in nature or exposed only to conditions designed to mimic fermenting cactus tissues in the wild.

Taken together, these data suggest that it is imperative that controlled choice experiments be conducted with flies with similar CHCs as those under conditions experienced by natural populations if we are to understand the pheromonal roles of CHCs in mating decisions between male and female *Drosophila*, as well as interpret how age-specific variation in CHCs influences sexual selection and sexual isolation. So far, no study has accomplished this, although one experiment employed wild-caught male flies in mating tests after they were exposed to laboratory food, and the CHCs of these males were found to significantly differ from those of laboratory food-reared flies (Hine et al. 2004). Further, desert-like conditions of low humidity and high temperatures in cactus-reared *D. mojavensis* caused significant shifts in different groups of CHCs (S. Rajpurohit, C. C. de Oliveira, W. J. Etges, and A. G. Gibbs, unpubl. data), so these factors should be examined as potential factors influencing mate choice. Now that experimental conditions are available to laboratory-reared flies with CHC amounts more similar to flies in nature, assessment of mating behavior, sexual selection, and sexual isolation needs to be repeated under these conditions with mainland and Baja California populations of *D. mojavensis*.

## Data accessibility

The cuticular hydrocarbon data are accessible at Dryad doi:10.5061/dryad.6s021.

## Supporting Information

**Figure S1**. Changes in amounts of the 16 most abundant CHCs with adult age in the population from Las Bocas, Sonora.

**Figure S2**. Changes in amounts of the 16 most abundant CHCs with adult age in the population from Punta Prieta, Baja California.

**Figure S3.** Male and female specific variation in PC 5 and PC 6 scores emphasizing age × sex and population × age interactions in amounts of adult CHCs with adult age.
