# Supplementary figures and images for: Premating isolation is determined by larval rearing substrates in cactophilic Drosophila mojavensis. X. Age-specific dynamics of adult epicuticular hydrocarbon expression in response to different host plants
Source: Ecol Evol. 2014 Apr 23;4(11):2033–45. doi: 10.1002/ece3.1088 (PMC4201419; doi:10.1002/ece3.1088)

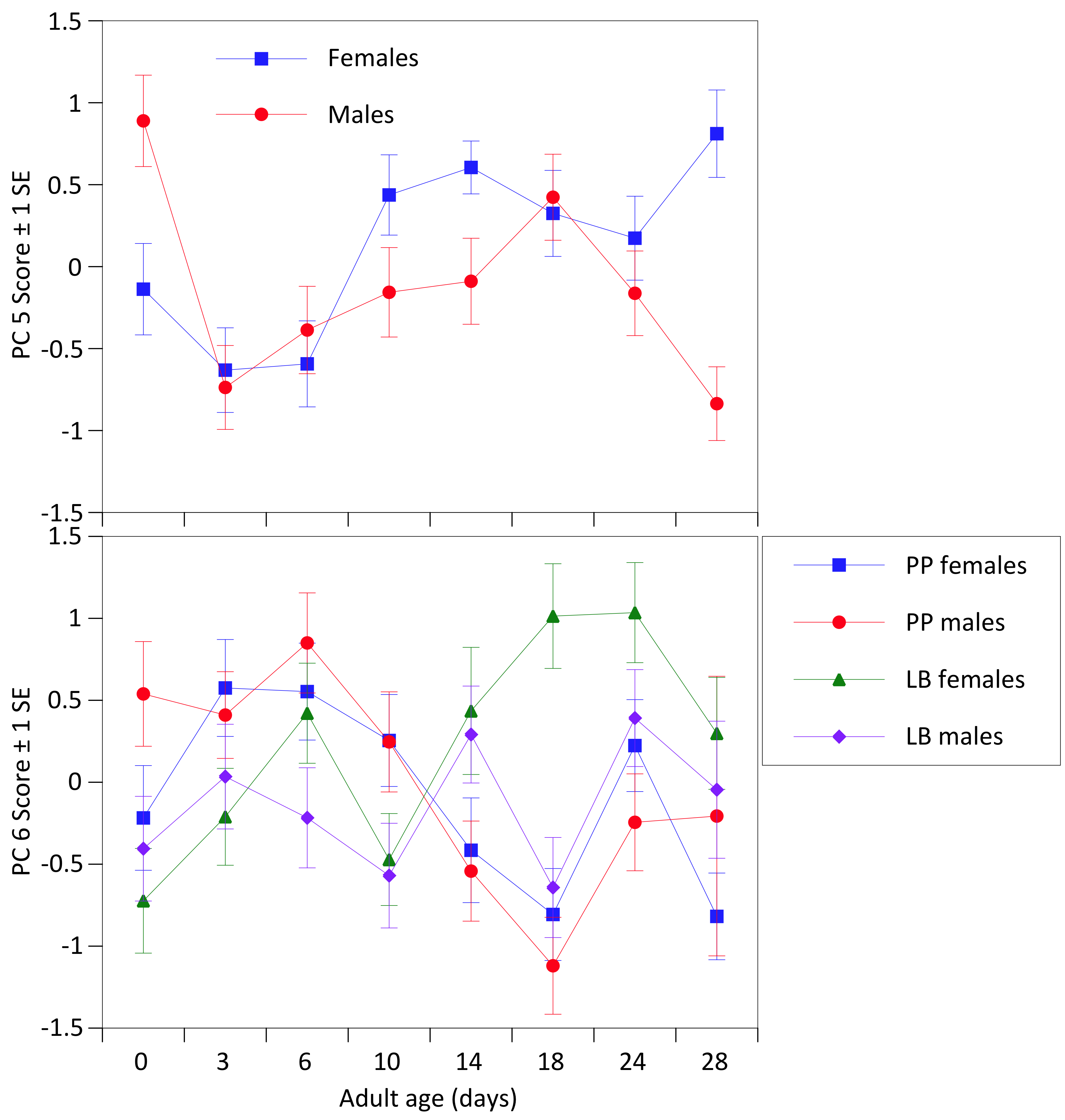

Supplement: Supplementary file 3 — Figure S3. Male and female specific variation in PC 5 and PC 6 scores emphasizing age × sex and population × age interactions in amounts of adult CHCs with adult age. [file ece30004-2033-sd3.docx]
